# Using Farm Animal Welfare Protocols as a Base to Assess the Welfare of Wild Animals in Captivity—Case Study: Dorcas Gazelles (*Gazella dorcas*)

**DOI:** 10.3390/ani8070111

**Published:** 2018-07-05

**Authors:** Marina Salas, Xavier Manteca, Teresa Abáigar, Maria Delclaux, Conrad Enseñat, Eva Martínez-Nevado, Miguel Ángel Quevedo, Hugo Fernández-Bellon

**Affiliations:** 1 Facultat de Veterinària, Universitat Autònoma de Barcelona, 08193 Bellaterra, Spain; xavier.manteca@uab.cat; 2 Estación Experimental de Zonas Áridas, Consejo Superior de Investigaciones Científicas, Crta.Sacramento, s/n, La Cañada de San Urbano, 04120 Almería, Spain; abaigar@eeza.csic.es; 3 Zoo Aquarium de Madrid, Casa de Campo, s/n, 28011 Madrid, Spain; MDelClaux@grpr.com (M.D.); emartinez@grpr.com (E.M.-N.); 4 Parc Zoològic de Barcelona, Parc de la Ciutadella, s/n, 08003 Barcelona, Spain; censenat@bsmsa.cat (C.E.); hfernandez@bsmsa.cat (H.F.-B.); 5 Zoobotánico Jerez, c/Madreselva, s/n, 11408 Jerez de la Frontera, Spain; miguel.quevedo@aytojerez.es

**Keywords:** behaviour, captivity, dorcas gazelle, enclosure, protocol, welfare, zoo

## Abstract

**Simple Summary:**

Animal welfare is gaining attention regarding the conservation of species not only due to ethical and legal reasons but also because optimal welfare can ensure stable and healthy populations. Currently, there is a lack of protocols that help to objectively assess welfare of wild animals in captivity. In this study, we have developed and applied a protocol for the assessment of welfare in captive dorcas gazelles (*Gazella dorcas*). We have gathered information from existing literature about the biology of this species in wild conditions, as well as in zoo husbandry, breeding, management and care guidelines developed for this species. We took a protocol developed for the on-farm welfare assessment in cattle as a reference and suggested 23 indicators that we considered useful to assess welfare in captive dorcas gazelles. To test the utility of this protocol, we then applied it in five groups of dorcas gazelles from three different zoos and we detected areas for improvement in all of the groups assessed.

**Abstract:**

There is a lack of protocols specifically developed for the assessment of welfare of wild animals in captivity, even when it is known that providing good standards of welfare is important. The aim of this study was the development and the application of a protocol for the assessment of welfare in captive dorcas gazelles. The protocol was mainly developed taking into account the protocol for the assessment of welfare in cattle from the Welfare Quality^®^ project, the available literature of the biology of this species and the Husbandry Guidelines developed for captive breeding and management of this species. The protocol was specifically developed for dorcas gazelles and included four principles, 10 criteria and 23 animal and environmental-based indicators. To test its utility, this protocol was applied to five different groups of gazelles from three different zoos. Its application made possible to detect areas for improvement in all groups assessed.

## 1. Introduction

Despite the ethical [1] and legal [2] importance of welfare in wild animals kept in captivity and that the insurance of optimal animal welfare is essential for the establishment and maintenance of viable populations of animals in good health [3], there is a lack of standardized and validated tools to assess welfare in captive animals.

Welfare assessment protocols can provide such tools by using a combination of several welfare indicators that provide information gathered by simple surveys, enclosure inspections and remote observation of animals. These protocols offer a simple, economic way to assess the welfare of captive animals, as well as to gauge the effect on animal well-being when an improvement is made.

The Welfare Quality^®^ project aims to develop tools for the objective assessment of the welfare of farm animals from a scientific point of view. As a result, objective protocols to assess welfare in cattle, poultry and pigs have been developed. These protocols are mainly based on animal-based measures, although they also have environmental or resource-based measures [4]. Animal-based indicators include variables that are measured directly in individuals. These are related to changes in the animals’ behaviour, overall appearance and health and include physiological parameters. On the other hand, resource-based indicators assess the environment surrounding the animal but not the animal itself (e.g., water provision, enclosure size and design and size and composition of a group or environmental enrichment).

Dorcas gazelles are part of the family Bovidae and are one of the smallest species of gazelles. These animals are naturally distributed around the Sahel-Saharan region (North Africa), living in a variety of habitats that include the savannah, semi-desert plains and desert areas [5]. They are ruminants; therefore, their diet is strictly herbivore and they eat succulent plants, hard desert herbs and fruits and leaves from different species of acacias [6]. They are gregarious animals with a strong hierarchical structure [7].

According to the International Union for Conservation of Nature (IUCN) Red List of Threatened Species [8], dorcas gazelles are considered vulnerable. Additionally, in 2002, the European Association of Zoos and Aquaria [9] established the European Endangered Species Program (EEP) for the subspecies Saharawi dorcas gazelle (**Gazella dorcas* neglecta*).

The aim of this project was the development and application of a protocol for the assessment of welfare of captive dorcas gazelles using the protocol for the assessment of welfare in cattle from the Welfare Quality^®^ [4] project as a base. The project was executed in two phases: first, the development of the welfare protocol; and second, the application of the protocol in centres holding dorcas gazelles.

## 2. Materials and Methods

The welfare protocol was developed using a combination of the study of dorcas gazelles’ biology and behaviour in natural conditions, the Husbandry Guidelines for the captive breeding and management developed specifically for this species [10] and the welfare protocols for cattle of the Welfare Quality^®^ project, since dorcas gazelles are also bovids.

In order to obtain general information about dorcas gazelle’s biology and behaviour, we used the Web of Science™ search engine using the keywords “dorcas gazelle*.” There is a lack of published scientific information about the biology of this species in the wild or about issues that they encounter in captivity. A total of 48 papers were reviewed and we found useful information to add to the protocol in four of the papers [6,7,11,12].

The Husbandry Guidelines for the captive breeding and management developed for dorcas gazelles provided information on current best practices and recommendations for the management of this species in captivity.

The Welfare Quality^®^ project protocols take into account four principles: good feeding, good housing, good health and appropriate behaviour as an expression of optimal emotional states. These four principles lead to 12 criteria that allowed for the development of welfare assessment indicators: absence of prolonged hunger and thirst; comfort around resting, thermal comfort and ease of movement; absence of injuries, diseases and pain induced by management procedures; and expression of social and other behaviours, good human-animal relationship and positive emotional state [13].

Once the welfare assessment protocol was developed, it was applied between May and June of 2013 to five groups of dorcas gazelles held in three centres participating in the EEP of the EAZA for this species: Parc Zoològic de Barcelona, Zoo Aquarium de Madrid and Zoobotánico Jerez. The five groups assessed are named as F (for ‘female group,’ *n* = 17), FY (for ‘female and young group’, *n* = 10) M1 (for ‘male group 1,’ *n* = 7), M2 (*n* = 3) and M3 (*n* = 5). The same person was responsible for the application of the protocol in all groups of dorcas gazelles and assessed the indicators following the instructions provided in the welfare assessment protocol (see below). The assessment of the animal-based indicators was done from the perspective of a zoo visitor without using any device such as binoculars.

## 3. Results

### 3.1. Development of the Welfare Protocol

The protocol developed for the welfare assessment of captive dorcas gazelles included four principles, 10 criteria and 23 indicators (Table 1). The 12 criteria of the Welfare Quality^®^ protocol were reduced to 10 criteria because two farm-related criteria were excluded. As additionally, the criteria ‘group size’ (that we considered essential for the welfare assessment in captive gazelles) was added. The criteria ‘positive emotional state’ was not added in this protocol because there is a lack of research on evaluation of emotions in zoo animals. A description of the 10 criteria, together with their proposed indicators, follows.

#### 3.1.1. Absence of Prolonged Hunger

Even though there are descriptions on other considerations related to feeding and diet requirements, no parameter related with this indicator is described in the Husbandry Guidelines for this species. However, body condition is included as an animal-based indicator in several protocols to assess welfare in animals. Poor body condition may be a consequence of inadequate nutrition, poor health, or chronic hunger; and it can have additional negative effects on health, behaviour and reproduction. Excessive body condition may increase the risk of lameness and other conditions and it may be a consequence of lack of physical exercise [14].

Both poor and excessive body condition are indicative of a welfare problem. However, currently there is no body condition scale developed for dorcas gazelles. Therefore, gazelles’ body condition (indicator 1.1.) is to be scored following the guidelines for the assessment of this indicator in another ruminant, the deer [15]. The animals are visually assessed from behind and from the side in the loin. Gazelles are scored as ‘Poor body condition’ under the following conditions: pelvis, ribs and spine prominent; concave rump area. ‘Normal body condition’ is evidenced by pelvis, ribs and spine not readily distinguished or appear rounded rather than sharp; rump area is flat. An animal seen as having ‘Excessive body condition’ has pelvis concealed by fat cover; rump very convex; spine not visible.

#### 3.1.2. Absence of Prolonged Thirst

Ad libitum access to good quality water is considered a welfare requirement. Welfare assessment protocols for farm animals include provision of water as a resource-based indicator. Gazelles should have easy access to drinking water area and troughs should be cleaned daily. The Husbandry Guidelines consider important that gazelles have ad libitum access to clean water changed daily.

The number of water points has to be checked (indicator 2.1.), as well as the availability of water (2.2.). The cleanliness of the water points (2.3.) with regard to the presence of old or fresh dirt on the inner side of the bowl or trough as well as staining of the water are also checked. Water points are considered clean when there is no evidence of crusts of dirt (e.g., faeces, mould), and/or decayed food residue, although some amount of fresh food is acceptable.

#### 3.1.3. Thermal Comfort

Even though dorcas gazelles are the most widespread of the gazelle species in the wild [6], in zoos they are often kept in climates that are very different from those of their natural habitat. However, it seems that gazelles can adapt to a diversity of climates. Nevertheless, wet and muddy conditions are likely to compromise welfare, as they may increase the risk of feet conditions. Very high temperatures might cause heat stress and sunburn if animals do not have access to shade. Currently, there is no precise information on the range of temperatures that is adequate for dorcas gazelles.

The Husbandry Guidelines describe that gazelles should have protection from bad climatic conditions, as well as from prolonged exposure to the sun. The guidelines also state that all enclosures should provide a shelter or stable with roof, enough vegetation or objects to provide shade, and, if possible, indoor facilities to have controlled temperature.

Two indicators were developed to assess the thermal comfort: availability of shade (indicator 3.1.) and shelter (3.2.). For the assessment of these indicators, it has to be recorded whether all animals in the enclosure can have access at the same time to a non-damp or non-muddy area, have adequate shade and have shelter from bad climatic conditions.

#### 3.1.4. Ease of Movement

Dorcas gazelles are found in a variety of habitats in the wild and can move fairly long distances depending on which habitat they live in [7]. Animals kept in small enclosures are more likely to develop physiological and behavioural changes indicative of poor welfare than animals kept in larger enclosures. Although the amount of space available for the animal is important, the quality and complexity of the space (e.g., whether there is any sort of environmental enrichment) is likely to be even more important [16]. Recommendations on the minimum space per animal vary, for which the rationale for such recommendations is not clear. According to the Husbandry Guidelines each gazelle should have a minimum of 46.5 m^2^ and 11.61 m^2^ for each additional animal. According to the American Association of Zoos and Aquariums [17], each gazelle should have a minimum of 18.6 m^2^. 

The enclosure size (area) (indicator 4.1.) and the square meters available per animal (4.2.) are two indicators developed to assess the ease of movement of dorcas gazelles in captivity. For the assessment of enclosure size, the area of the enclosure has to be calculated. For the determination of the square meters available per animal the area of the enclosure is divided by the total number of animals found in that enclosure. Also, it has to be recorded if there are adequate resting places for all the animals at the same time.

#### 3.1.5. Absence of Injuries

Two indicators were developed to assess the absence of injuries: lameness and integument alterations.

Hoof problems are common in Artiodactylds [18]. Along with other feet problems such as traumas due to aggressive behaviours, consolidated bone fractures or other conditions can lead to lameness. In farm animals, lameness is considered a major welfare problem as it is indicative of pain and may interfere with normal behaviour.

Animals are observed in motion because lameness (indicator 5.1.) is an abnormality of gait that is more evident when the legs are in motion. Animals have to be scored as ‘not lame’ (when the animal walks without any apparent abnormality) or ‘lame’ (when the animal walks with an apparent abnormality or without resting one or more legs on the floor).

Integument alterations such as hairless patches and lesions or swellings may be a consequence of disease, rough handling, intra-specific aggression, or inappropriate physical environment. In farm animals, presence of injuries on the integument is commonly used as an indicator of poor welfare. The Husbandry Guidelines recommend paying attention to objects, handling procedures and other animals (especially when intra-specific aggressive behaviours have been observed in enclosures shared with other species) that could cause lesions to the gazelles.

Only skin alterations (indicator 5.2.) of a minimum diameter of 2 cm at the largest extent have to be counted. A hairless patch includes an area with hair loss, with the skin not damaged, an extensive thinning of the coat due to parasites and hyperkeratosis. A lesion/swelling includes damaged skin either in form of a scab or a wound, dermatitis due to ectoparasites and ear lesions due to torn off ear tags. Without touching the animals, three body regions on one side of the assessed animal have to be examined: body, hind leg and front leg. These body regions are scanned from the rear to the front, excluding the bottom side of the abdomen and the inner side of the legs but including the inner side of the opposite hind leg. Random side selection (right or left) before the examination has to be ensured, in order to prevent biased results.

Animals are scored as follows: ‘no integument alterations’; ‘mild integument alterations’ meaning there is at least one hairless patch but no lesion/swelling; and ‘severe integument alterations’ meaning at least one lesion/swelling or large hairless patch.

#### 3.1.6. Absence of Disease

According to the Husbandry Guidelines, the most frequent diseases or afflictions in captive dorcas gazelles are traumatisms, behavioural disorders, gastrointestinal and respiratory affections and birth problems. Traumatisms are due to fights, accidents at capture or management, or by accidental trampling by larger species. These can be assessed through the inspection of integument and if the animal exhibits lameness. 

The Welfare Quality^®^ protocols for ruminants include some indicators that can be used as a tool to assess (through remote observation) the gastrointestinal and respiratory affections described in the Husbandry Guidelines. 

Nasal discharge (indicator 6.1.) is when “clearly visible flow/discharge from the nostrils that can be transparent to yellow/green and often is of thick consistency” Ocular discharge (6.2.) is when “clearly visible flow/discharge (wet or dry) from the eye, at least 3 cm long.” Hampered respiration rate (6.3.) is “deep and overtly difficult or laboured breathing; expiration is visibly supported by the muscles of the trunk, often accompanied by a pronounced sound.” Diarrhoea (6.4.) is a “loose watery manure below the tail head on both sides.” Animals are scored with regard to each indicator as ‘no evidence’ or ‘evidence’ of the specific indicator.

#### 3.1.7. Expression of Social Behaviours

Affiliative behaviours are considered self-rewarding and may have a buffering effect on stress. However, in domestic cattle, social grooming may increase after stressful events. The Husbandry Guidelines do not include information regarding affiliative behaviours among adult animals.

Intra-specific aggression may lead to injuries and social stress and has been included in several protocols to assess welfare in farm animals. According to the Husbandry Guidelines, adult males of dorcas gazelles usually present high levels of aggression while in captive conditions. It is also usual for a breeding male to attack other males when moved from a reproductive group to a bachelor group. Some degree of intra-specific aggression may be normal or even unavoidable, therefore, only ‘excessive’ aggression should be indicative of poor welfare. Currently there is no definition of ‘excessive’ intra-specific aggression in dorcas gazelles. However, presence of overt aggression and frequent threats between animals should be considered indicators of poor welfare.

Two indicators were developed to assess the expression of social behaviours: affiliative behaviours (indicator 7.1.) and intra-specific aggression (7.2.). An ethogram with the social behaviours is included in Table 2. Overall observation time is 180 min per group, in nine 20-min long sessions. The frequency of social behaviours has to be recorded using continuous focal behaviour sampling, since social behaviours may be subtle and of short duration.

#### 3.1.8. Group Size

Three indicators were developed to assess the group size, including number of gazelles in the group, composition of the group and number of animals of other species.

Dorcas gazelles have a complex and habitat-related social organization. The different social structures are largely a consequence of the availability and distribution of food resources: dorcas gazelle group size increases with increased forage quality [7,12]. Four situations have been observed in the wild: harem-like structure (social units with one male accompanied by one-five females), satellite groups of immature males, female herds unaccompanied by males and male pairs. In zoos, animals are usually kept in four groups: females and young with only one reproductive male; females and young without adult males; bachelor groups of males; and isolated males (usually the reproductive males of a harem).

Social contact is necessary for good welfare in group-living animals and social isolation has been associated with stereotypies, chronic stress and incompetence in regard to reproductive and social behaviours [19]. In domestic social species, being kept in groups is considered a requisite for good welfare.

Group size is not the only factor to consider, as group composition and compatibility between individual animals are also important. The Husbandry Guidelines propose approximate numbers of how big captive groups should be; however, the rationale for such recommendations is not clear. The Husbandry Guidelines suggest one adult male and 3–7 adult females and their young in reproductive groups and between 3 and 7 adult males in bachelor groups. A number in the case of the group with adult females and young is not specified. Only aggressive adult males should be kept isolated if a treatment with long-action tranquilizers does not work. Females should not be kept isolated because they may experience increased levels of stress.

For the assessment of the number of gazelles in the group (indicator 8.1.) and composition of the group (8.2.), how many animals form the group is assessed along with their age and sex. Also recorded are if any individual (regardless of age, sex, or if it is castrated or not) is kept alone without any contact from other conspecifics. 

The current tendency in zoos is to make larger and more naturalistic enclosures [20]. Mixing species that share the same space is done as an enrichment source sometimes. However, this must be done carefully. In the case that dorcas gazelles share their enclosure with individuals of species bigger than them, the Husbandry Guidelines recommend the use of selective gates that only gazelles can go through. Gazelles could use those gates to evade or avoid individuals from another species.

When assessing the number of animals of other species (indicator 8.3.), the number of animals of each species whom share the enclosure with dorcas gazelles is recorded. Any information related to the social inter-specific interactions or the use of space provides valuable information for welfare assessment.

#### 3.1.9. Expression of Other Behaviours

Stereotypies were defined as behaviours that are repetitive, invariable and without any apparent function. More recently, stereotypies have been described as repetitive behaviours induced by frustration, repeated attempts to cope, and/or due to dysfunctions in the central nervous system [21,22]. Some stereotypies might help animals cope in difficult environments [23,24]. In general, stereotypies are considered to be indicators of a lack of good welfare [25]. This is due to both the circumstances that favour their development, such as restrictive environments that prevent the expression of normal species-specific behaviours and the fact that some stereotypies negatively affect the animal, causing injury or loss of body condition [22,26].

The Husbandry Guidelines do not contain any information regarding stereotypies in dorcas gazelles. However, although stereotypies are not widely reported in this species, ungulates are particularly at risk of developing oral stereotypies in captivity [27]. In fact, repetitive, seemingly functionless oral and oro-nasal activities (e.g., object-licking, dirt-eating, tongue-rolling, etc.) are prevalent in captive ungulates [28].

The relationship between stereotypies and individual welfare is complex. Stereotypies may persist even after the environment in which the animal is kept has been considerably improved. Additionally, poor welfare status may not always result in developing stereotypies [29]. Stereotypies can also appear as a result of learning, in which case they are not suitable welfare indicators [25].

All the animals should be assessed for the presence or absence of stereotypic behaviours (indicator 9.1.) during the observations of the expression of social behaviours. Animals are scored as ‘no presence of stereotypic behaviour’ or ‘presence of stereotypic behaviour.’ If any stereotype is recorded, the behaviour must be described.

Environmental enrichment has positive effects on welfare in captivity. Dorcas gazelles in the wild use various kinds of trees for different purposes [11]. Larger acacia trees are used for territorial purposes because gazelles use middens (or dung piles) for activities related to territory maintenance, advertisement and olfactory communication. Larger trees also provide more shade, another food source (such as seed pods) and cover from predators than smaller trees. The loss of large trees in the wild may indirectly affect social behaviour of dorcas gazelles because animals are losing conspicuous landmarks that could be used for midden sites. Dorcas gazelles in the wild spend much of their total time foraging and browsing and shorter trees are a source of browsing material. 

The Husbandry Guidelines consider it essential that gazelles have the opportunity to perform natural behaviours for the species, as well as grazing, browsing, marking the territory, keeping a healthy physical condition and running away or hiding. With that aim, they recommend the use of structural components, such as rocks, vegetation, irregular ground, to change the feeding routine, or to provide sensorial stimulation with new sounds or smells.

For measuring the environmental enrichment program (indicator 9.2.) it must be recorded whether or not the animals are given access and opportunities to browse and if there are trees/poles/sticks/post/other vertical objects of different form and size. If the centre has an enrichment program established for dorcas gazelles, it should be described which enrichments are used and how often the enrichment material is changed.

#### 3.1.10. Good Human-Animal Relationships

If properly done, medical training is likely to reduce the stress caused by veterinary procedures. Training based on positive reinforcement could have positive effects on welfare and be considered as a form of enrichment. Poor training techniques such as training based on punishment, or carried out by inexperienced staff, have negative effects on welfare and are not recommended. If medical training is not possible, handling systems that minimize stress during veterinary interventions are recommended.

For measuring the medical training (indicator 10.1.) it should be recorded whether or not the centre uses a medical program for this species, if it is based on positive or negative reinforcement techniques and if the training is carried out by experienced staff.

The Husbandry Guidelines do not mention the implementation of a medical training program, although it recommends that the capture, immobilization and handling of the animals is intended to cause as little stress as possible.

The methods used to capture, immobilise and handle the animals (indicator 10.2.) must also be recorded.

### 3.2. Application of the Welfare Protocol

The results obtained after the application of the protocol are as follows:

#### 3.2.1. Absence of Prolonged Hunger

The application of the protocol for body condition (indicator 1.1.) showed that all animals assessed had a ‘normal body condition.’

#### 3.2.2. Absence of Prolonged Thirst

The assessment of the water points (indicator 2.1.) for group M1 was not possible. Groups F and FY had three water points and groups M2 and M3 had two.

The water point quality (both the presence of water (indicator 2.2.) and cleanliness of the troughs (2.3.) should be improved in all centres assessed; especially in group FY. In this group, of the three water points present, only two had water and the water was dirty in both. Only two groups had at least one clean water trough (groups M2 and M3), all the others were dirty or partly dirty.

#### 3.2.3. Thermal Comfort

Animals of groups F, FY and M1 had shade (indicator 3.1.) and shelter from bad conditions (3.2.) available to all the animals at the same time. Gazelles in group M2 shared the enclosure with two white rhinoceros (*Ceratotherium simum*). During the hottest hours of the day there was only one source of shade available, provided by an acacia next to the enclosure. The shade was exclusively used by the rhinoceros and therefore the gazelles were continuously exposed to the sun. They also did not have shelter from bad conditions. Group M3 also did not have shelter from bad conditions, although the zoo location has a low frequency of precipitation, so rain and storms are not very common. In this case, a shelter from bad weather might not be as important as in other locations.

#### 3.2.4. Ease of Movement

The area (indicator 4.1.) and the square meters available per animal (4.2.) were different in all groups (Table 3). Group M2 had one of the largest enclosures and the enclosure with more space per animal. However, this enclosure offered the least amount of shelter from bad conditions and direct sun. There were also less trees or vertical objects for gazelles to use for territorial purposes and social communication. All groups fulfilled the minimum space per animal requirement according to the Husbandry Guidelines.

#### 3.2.5. Absence of Injuries

One animal from group M2, one from group M3 and two from group FY were scored as ‘lame.’ All the lameness had been previously documented in the veterinarian records of each institution and no new lameness (indicator 5.1.) was detected during the application of the protocol.

The assessment of integument alterations (indicator 5.2.) showed that none of the animals presented lesions, swellings or large hairless patches in their integument. However, due to the difficulty to observe the animals at a closer distance, it was not possible to look for small hairless patches in every animal.

#### 3.2.6. Absence of Disease

None of the animals observed had nasal or ocular discharge, hampered respiration or diarrhoea (indicators 6.1., 6.2., 6.3. and 6.4., respectively) during the application of the protocol.

#### 3.2.7. Expression of Social Behaviours

**Figure 1:**
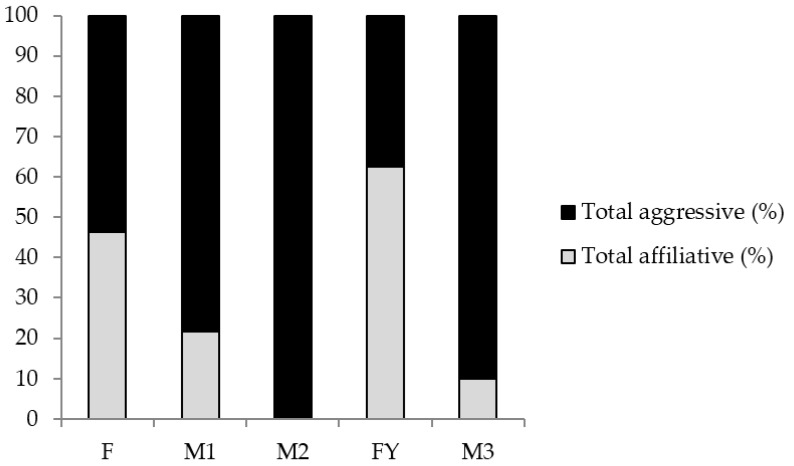
Percentage of aggressive and affiliative behaviours out of the total number of social interactions recorded for each group of dorcas gazelles studied (*n* = 42). The five groups assessed are named F (for ‘female group,’ *n* = 17), FY (for ‘female and young group’, *n* = 10) M1 (for ‘male group 1,’ *n* = 7), M2 (*n* = 3) and M3 (*n* = 5).

The application of the protocol for the indicators affiliative behaviour (7.1.) and intra-specific aggression (7.2.) showed that male groups (M1, M2 and M3) displayed more aggressive behaviours than the female (F) and female with young groups (FY) (Figure 1).

#### 3.2.8. Group Size

When assessing the number of gazelles in the group (indicator 8.1.) and the composition of the group (8.2.), it was observed that none of the zoos kept gazelles individually and that all animals were part of a social group (F, *n* = 17 (all females); FY, *n* = 10 (four females and six sub-adult or young with less than 10 months of age); M1, *n* = 7 (all males); M2, *n* = 3 (all males); and M3, *n* = 5 (all males)). Also, no mature males were sharing the same space with a group of adult females. 

When assessing the number of animals of other species (indicator 8.3.), it was observed that, with the exception of group F, all groups shared their enclosures with other species. Although group M1 shared its space with five scimitar-horned oryx (*Oryx dammah*), group FY with three common ostriches (*Struthio camelus*) and group M3 with two Rothschild’s giraffes (*Giraffa camelopardalis rothschildi*), no inter-specific agonistic interactions were observed. As previously stated, the gazelles of group M2 shared space with two white rhinoceros that occupied the only shade available in the enclosure during the hottest hours of the day, therefore the gazelles were subjected to more hours of sun exposure. Additionally, all enclosures assessed where gazelles shared the space with other species had gates which allowed them to hide.

#### 3.2.9. Expression of Other Behaviours

None of the animals observed presented any kind of stereotype (indicator 9.1.).

No formal enrichment program (indicator 9.2.) was implemented at any zoo. None of the animals were provided with browsing material regularly, although in all centres browsing material for the animals were provided at some point. However, this was sporadic and not scheduled. A good enrichment program is strongly recommended in all the zoos, with special opportunities to browse regularly. Group M2 had only three vertical objects but all of them were of the same size and material.

#### 3.2.10. Good Human-Animal Relationships

None of the groups of dorcas gazelles assessed was part of a medical training program (indicator 10.1.).

The capture, immobilization and handling of the animals (indicator 10.2.) varied from one centre to another. In groups FY, M1 and M2 the capture of the animals was performed in dark, indoor stables where the animals usually lay down, making the capture easier. In groups F and M3 a net was used to capture the animals. The posterior immobilization and handling of the animals was generally the same in all groups with the eyes of the gazelles covered to decrease stress and their legs firmly held to avoid self-inflicted harm due to escape attempts.

## 4. Discussion

Animal welfare is comprised of the emotional state, physical health and behaviour of the animals. There is no single indicator capable of providing enough information to thoroughly assess animal welfare, therefore it can only be appropriately assessed when using a combination of several indicators [13]. However, even if many indicators are needed, it is important to develop protocols for easy application. 

In our protocol, we found some indicators easier to apply than others. Specifically, we encountered significant difficulties while assessing the integument alterations (indicator 5.2.) in some animals. While it would be easy to detect large hairless areas, lesions, or swelling in the skin, it was very difficult to detect small hairless patches in every animal from afar. We do not consider this fact excludes the indicator as being useful, as the assessment of integument alterations is imperative in a welfare protocol. This is especially important in species where frequent aggressive interactions have been observed, such as dorcas gazelles [10,30]. The use of devices such as binoculars would be necessary in order to properly assess this indicator.

We deemed it important to add the assessment of medical training (indicator 10.1.), since, if done properly, it is likely to reduce the stress caused by certain veterinary procedures. Modern zoos have mastered the use of training techniques and medical training programs. These are applied frequently in species such as elephants, giraffes, big cats and apes. However, their application in species such as gazelles are not very common. This, among other reasons could be due to the risk involved in working with predators or larger species, compared to gazelles. This makes the implementation of medical training programs a priority in species with a more difficult husbandry. Nevertheless, gazelles would benefit from training programs that make veterinarian interventions less stressful. With proper training, the need to capture and restrain individuals would be unnecessary and would avoid injuries and issues related to the capture.

As mentioned earlier, the current zoo trend is to make larger and more naturalistic enclosures [20]. This often includes the mixture of different species, which can be beneficial for the animals as an enrichment source and adds educational value for the visitors [31]. However, if there are different species sharing the same enclosure, we should make sure all animals have adequate access to all the available resources. For example, it was observed that one of the groups of gazelles assessed shared the enclosure with two white rhinoceros that occupied the only shade available in the facility during most of the daytime. This left the gazelles exposed to direct exposure to the sun for long periods of time. Also, in this same enclosure there was a lack of vertical objects that animals use for communication purposes [11]. Along with creating more shade, the addition of trees, posts, or other vertical objects into the enclosure would give the animals more opportunities to communicate, thereby solving two shortcomings with only one action.

Contrary to the Welfare Quality^®^ protocols for farm animals, in our protocol we did not include the criteria ‘positive emotional state.’ The focus of animal welfare science is leading towards the experience of positive states or to minimize negative experiences while creating more opportunities to have positives ones. These positive states include comfort, pleasure, interest, confidence, or sense of control [32]. This criterion was not included in the protocol because there is still not enough research done on zoo animals in relation to this subject. However, positive emotional state is an important criteria that should be taken into consideration for future protocols when assessing wild animals in captivity.

This welfare protocol developed for dorcas gazelles is the first documented work towards developing a standardized welfare assessment tool for this species. This is not the first welfare protocol for wild animals in captivity that has been developed using the Welfare Quality^®^ protocols as a reference. An on-farm welfare assessment protocol for foxes (*Vulpes* spp.) and mink (*Neovison vison*) [33] was developed by adapting the Welfare Quality^®^ framework to a novel species. Clegg et al. [34] also presented their work about the development of a welfare assessment index for captive bottlenose dolphins (*Tursiops truncatus*).

The ultimate goal of a protocol to assess welfare in wild animals kept in captivity is to have it used regularly as a management tool in centres where these animals are held. A successful protocol should be practical and easy to apply. The protocol developed for the assessment of welfare in bottlenose dolphins [34] takes an estimated two days for the evaluation of a group of 10 individuals. The protocol developed for foxes and mink [33] requires three visits to each farm, which will cover the lifespan of all animals. This protocol admits that its implementation is challenging in practice. The largest group of dorcas gazelles assessed in this study was comprised of 17 individuals and all the indicators were evaluated in less than six hours per group.

This protocol is presented as an initial phase to assess the welfare of captive dorcas gazelles and validation has not been completed. However, its application in five participant groups of the EEP of the EAZA for this species and the analysis of the results allowed the detestation of some areas for improvement in all the centres assessed. 

## 5. Conclusions

We developed a protocol for the assessment of welfare in dorcas gazelles in captivity taking a protocol for the assessment of welfare in cattle as a base. The protocol, specifically developed for dorcas gazelles, included four principles, 10 criteria and 23 animal and environmental-based indicators. After the application of the protocol to five different groups of gazelles it was possible to detect areas for improvement in all groups. Therefore, we believe this protocol can be a useful tool for those centres that keep dorcas gazelles in captivity.

## Figures and Tables

**Table 1:** Principles, criteria and indicators of the protocol to assess welfare in captive dorcas gazelles. There are 10 animal-based indicators (indicated by ‘*’) and 13 resource or management-based indicators.

Principles	Criteria	Indicators
Good Feeding	Absence of prolonged hunger	1.1. Body condition *
	2. Absence of prolonged thirst	2.1. Number of water points 2.2. Availability of water 2.3. Cleanliness of the water points
Good Housing	3. Thermal comfort	3.1. Availability of shade 3.2. Availability of shelter
	4. Ease of movement	4.1. Enclosure size (area) 4.2. Square meters available per animal
Good Health	5. Absence of injuries	5.1. 1 Lameness * 5.2. Integument alterations *
	6. Absence of disease	6.1. Nasal discharge * 6.2. Ocular discharge * 6.3. Hampered respiration * 6.4. Diarrhoea *
Appropriate Behaviour	7. Expression of social behaviours	7.1. Affiliative behaviour * 7.2. Intra-specific aggression *
	8. Group size	8.1. Number of gazelles 8.2. Composition of the group 8.3. Number of animals of other species
	9. Expression of other behaviours	9.1. Stereotypies * 9.2. Environmental enrichment program
	10. Good human-animal relationship	10.1. Medical training program 10.2. Capture, immobilization and handling

**Table 2:** Description of the social behaviours that are included in the welfare protocol for dorcas gazelles.

Affiliative behaviour	**Behaviour**	**Definition of the Behaviour**
	Social grooming	The animal brushes with its muzzle any part of the body of another group mate except for the anal region or the prepuce. If the animal stops brushing the receiver for more than 10 s and then starts brushing the same receiver again, this is recorded as a new bout. It is also taken as a new bout if the actor starts brushing another receiver or if there is a role reversal between actor and receiver.
	Social smelling	The animal smells any part of the body of another group mate except for the anal region or the prepuce. If the animal stops smelling for more than 10 s and then starts smelling the same receiver again, this is recorded as a new bout. It is also taken as a new bout if the actor starts smelling another receiver or if there is a role reversal between actor and receiver.
	Horning	Head play with physical contact of two animals. The animals rub foreheads, horn bases or horns against the head or neck of one another without obvious agonistic intention. Neither of the opponents takes advantage of the situation in order to become victorious. It is taken as a new bout if the same animals start horning after 10 s or more, or if the horning partner changes.
Aggressive behaviour	Displacement with physical contact	Interaction where the actor is butting, hitting, thrusting, striking, pushing or penetrating the receiver with forehead, horns, horn base or any other part of the body with a forceful movement resulting in the receiver giving up its position.
	Displacement without physical contact	The actor threatens or interacts with the receiver without making any physical contact resulting in the receiver giving up its position.
	Chasing	The actor makes an animal flee or give up its current position by following fast or running behind it, sometimes with additional threats like jerky head movements. Chasing is recorded even if it is not followed by an interaction with physical contact.
	Fighting	Two contestants vigorously push their heads (foreheads, horn bases and/or horns) against each other while planting their feet on the ground, both exerting force against each other. A new bout starts if the same animals restart fighting after more than 10 s or if the fighting partner changes.

**Table 3:** Enclosure size (area) and space per animal available in each group of dorcas gazelle assessed.

Group	Number of Animals	Enclosure Size (Area) (m^2^)	Space per Animal (m^2^/Animal)
F	17	728	43
FY	10	1143	88
M1	7	1117	93
M2	3	1143	229
M3	5	1287	184
